# Nanobubbles in Electrospray Ionization Mass Spectrometry

**DOI:** 10.1021/acs.analchem.4c06040

**Published:** 2025-03-10

**Authors:** George Joseph, Bincy Binny, Andre R Venter

**Affiliations:** Department of Chemistry, Western Michigan University, Kalamazoo, Michigan 49008, United States

## Abstract

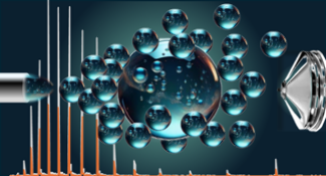

Nanobubbles (NBs) are very small gas-filled cavities in solvents, and when their sizes reach diameters around 200 nm, they remain in solution for extended periods of time, featuring special chemical and physical properties. Here, we investigate the application of NBs in electrospray ionization. We show that the addition of CO_2_ or N_2_ NBs into spray solvents significantly improves signal responses of small molecules in both positive and negative modes during ESI-MS. The magnitude of the increase depends on analyte identity, solvent system, NB gas composition, and the method for preparing the NBs. When NBs are used to analyze proteins, both signal intensities and charge states increase. This is tentatively attributed to NB’s increasing the total area of the hydrophobic gas–liquid interface, on which proteins can unfold, and improved transport of analytes to the droplet surface. This increase in the interface is likely also a contributing factor in the further enhancement of the rate at which reagents are converted into products when NBs are present compared to those measured from accelerated reactions from microdroplets during reactive-ESI experiments. Moreover, NBs can overcome solubility barriers when one of the reagents is gaseous and, thus, can be incorporated into an NB. This was demonstrated in the reaction between *N*,*N*-dibutyl-1,3-propane diamine and carbon dioxide, where the reaction conversion rate could be significantly improved when N_2_ NBs were present in solution, but even more so when the bubbles were composed of CO_2_.

## Introduction

Electrospray ionization mass spectrometry (ESI-MS) is a useful tool for the analysis of small to very large polar molecules and is essential in proteomics,^1^ metabolomics,^2^ lipidomics,^3^ and numerous other fields.^4^ Additives such as formic acid, ammonium acetate, and ammonium bicarbonate are frequently used as part of the sample working solution, to increase conductivity, to aid in forming protonated or deprotonated species, or to regulate or supply other charge-carrying ionic species, to increase or decrease charge states of analytes such as proteins, etc.^5,6^

The creation or existence of bubbles during ESI has been hypothesized to explain various observations, such as electrothermal supercharging of protein molecules,^7^ increased reaction rates of amines with carbon dioxide during reactive ESI,^8^ and improved desorption efficiency of proteins by desorption electrospray ionization (DESI-MS) when ammonium bicarbonate is used as an additive.^9^ In this study, we investigate a novel type of additive, the deliberate addition of solution-stable nanobubbles (NBs), to the spray solution during ESI-MS. Our results, to a large extent, confirm the previously inferred presence and benefits of bubbles in the aforementioned reports.

Bulk NBs are very small gas-filled cavities in solution that exhibit special qualities when their diameters approach around 200 nm in water. Unlike microbubbles, bubbles of this size and smaller do not coalesce to burst at liquid surfaces but can remain stable in solution for extended periods of time.^10^ NBs are known to have a strong affinity for hydrophobic surfaces with extra-large wetting angles, may produce free radicals, possess high internal pressure, and provide an enormous surface-to-volume ratio, which provides a high gas dissolution capacity. An important property of NBs is the surface electrical charge, which is expressed by zeta potential. Zeta potential values are gas- and solvent-dependent and are usually negative. In neutral water, O_2_ and N_2_ NBs have zeta potentials of −34 to −45 and −29 to −35 mV, respectively, while CO_2_ NBs have reported zeta potentials of −17 to −36 mV.^11^ The negative zeta potential of NBs leads to the formation of an electrical double layer contributing to the extended lifetimes of NBs by inhibiting coalescence.^12^ These characteristics make NBs very promising for use in a wide range of cutting-edge scientific applications^13−15^ and are important in fields such as wastewater treatment,^16^ surface cleaning,^17^ drug delivery,^18^ and tumor destruction.^19^

In this work, we demonstrate that it is easy to generate CO_2_ and N_2_ NBs in ESI spray solutions using laboratory-scale batch processing methods. The methods of NB generation included the previously established methods of sonication^20^ and pressure cycling,^21^ as well as a novel method based on flow regime switching using a Tesla valve (Figure S1).^22,23^ We investigate the beneficial effects and other consequences of using NBs as spray additives in ESI-MS on spray stability and signal response of proteins, lipids, and small molecules in both positive and negative modes.

## Experimental Section

### Materials

Pure ethyl alcohol (99.5%) was obtained from Sigma-Aldrich (St. Louis, Missouri). Milli-Q water (18 MΩ cm^–3^) was obtained from a Thermo-Barnstead Water Polisher (Thermo Scientific, Waltham, MA, USA). Ammonium bicarbonate (ABC, BioUltra grade), bovine heart cytochrome C (Cyt c, 95% purity), formic acid (FA, ≥98%), hydrocortisone, *N*,*N*-dibutyl-1,3-propane diamine (DBPA), caffeine, and ibuprofen were purchased from Sigma-Aldrich (St. Louis, MO). Pure Rhodamine 6G (99%) was purchased from Acros Organics (New Jersey, USA). International calibration extract 4 (ICE 4) standard hops extract was purchased from American Society of Brewing Chemists (St. Paul, Minnesota).

### Sample Preparation

Protein stock solutions were prepared at 160 μM in Milli-Q water with 1.0 M ABC. Caffeine, hydrocortisone, and ibuprofen stock solutions were prepared in 100% methanol at 10 mM. Hydrocortisone and ibuprofen stock solutions were prepared in 100% methanol. These stock solutions were further diluted to the indicated concentrations in solvent systems that contained 50% MeOH and either water as control or NB-enriched water. Some experiments also contained 0.2% FA or 100 mM ABC as indicated. For the ion suppression experiments, Rhodamine 6G was kept at a constant concentration of 1 μM, while the caffeine concentration was varied from 97 nM to 100 μM. A standard solution of ICE 4 hops was prepared by dissolving 0.5 g of the extract in 1.0 mL of methanol. The vial was then vortexed until the extract was completely dissolved. The stock solution was diluted 250 times in 95% MeOH with and without CO_2_ NB-enriched water (for an ESI-ready standard, a total concentration of 14 ng/L hops acids).

For microdroplet reaction experiments, a stock solution of 10 mM DBPA was prepared in 100% ACN. A 10 μM DMPA solution was prepared by using 95% ACN with and without CO_2_ or N_2_ NBs in the 5% water fraction.

### Nanobubble Generation

Carbonation of 3% ethanol solution was obtained using a SodaStream Terra sparkling water maker (Mount Laurel, New Jersey, USA). Similarly, 3% ethanol solution was sparged with 99.999% N_2_ (Airgas, Gwinnett, Georgia) for 5 min at a tank pressure of 50 psi and volumetric flow rate of 5.8 L/min.

CO_2_ and N_2_ NBs were generated by flow regime switching using a valvular conduit. This device was invented by Nikola Tesla in 1920 and is commonly known as the Tesla valve.^24^ The valve can induce turbulent flow at lower Reynolds numbers in one direction while it is laminar in the opposing direction due to the nonsymmetrical flow paths in the opposing directions.^25^ A 3D-printed Tesla valve with a length-to-depth ratio of 21 was used for generation of NBs. The Tesla valve was printed using a Phrozen Sonic Mini 8K LCD Resin 3D printer using a Siraya Tech Blu 3D printer resin (San Gabriel, California). The print file was based on a plan published by Bao et al.^26^ Carbonated 3% ethanol solution was used for the generation of CO_2_ NBs, while N_2_ NBs were generated from N_2_-saturated solutions. NB generation was optimized to 12 cycles. One cycle comprises a forward and backward directional flow, as shown in Figure S1.

NBs were also generated by pressure cycling^21^ and sonication methods.^27^ With the pressure cycling method, NB generation was optimized to 60 cycles when using 5 mL of carbonated solution in a 10 mL polypropylene syringe. For NB production by sonication, the previously optimized value of 5 min at a frequency of 10 kHz was used. NB generation and size distributions were obtained by dynamic light scattering (DLS) (DynaPro Titan, Wyatt Technology Corporation, Santa Barbara, CA), and NB numbers and zeta potentials were determined by a PMX-230 TWIN Laser Zeta View Nanoparticle Tracking Analysis system (NTA) (Particle Metrix, Ammersee, Germany). The weighted average diameters, bubble concentrations, and zeta potentials measured by NTA are provided in Table 1.

**Table 1:** Physical Characterization of CO_2_ Nanobubbles under the Optimized Conditions for Each Nanobubble Generation Method by Nanoparticle Tracking Analysis

	weighted average size		average concentration		
method	diameter (nm)	rel. std. dev. (%)	concentration (NBs/mL)	rel. std. dev. (%)	zeta potential (mV)
pressure cycling	143	3.6	2.61 × 10^9^	19.2	–21.27
sonication	99	11.0	2.99 × 10^8^	14.0	–36.17
Tesla valve	110	8.3	3.79 × 10^8^	7.4	–33.30

### Mass Spectrometry

Experiments were performed on an LTQ linear ion trap mass spectrometer (Thermo Scientific, Waltham, MA). Electrospray ionization was achieved using a home-built microelectrospray emitter made from a Swagelok T-piece and two pieces of coaxial fused silica capillary tubing. The outer capillary (for sheath gas) was approximately 20 mm in length with an outer diameter of 360 μm and an inner diameter of 250 μm. The internal capillary (for solvent) had an outer diameter of 150 μm and an inner diameter of 50 μm. The solvent capillary extended through the T-piece and was connected to a syringe pump, which delivered the working solutions for direct infusion. Spray potential was applied to the liquid junction of a stainless-steel syringe needle, which delivered the solvent at a flow rate of 5 μL/min, with N_2_ as the nebulizing gas at 100 psi. LTQ capillary voltage and tube lens voltage were set at 30 and 135 V for the analysis of cytochrome c, 20 and 65 V for caffeine, 20 and 65 V for hydrocortisone, 20 and 60 V for ibuprofen, and −20 and −65 V for hops acid, respectively. The spray potential was ±4.0 kV, except for the hops acids where −3.3 kV was used.

### Data Analysis

Mass spectra were collected and viewed in Xcalibur Qual Browser (2.0.7). Thirty scans of direct infusion of three independent samples were collected for each solvent system of protein, caffeine, and ibuprofen.

## Results and Discussion

### Nanobubble Generation and Characterization

NBs were produced using the three different methods of flow regime switching with a Tesla valve, pressure cycling, and sonication in 3% ethanol solution. The presence of NBs was confirmed by DLS and NTA. 3% ethanol has been shown to increase bubble stability and reduce bubble diameters.^28^ The obtained size distributions by DLS are shown in Figure S2, and the NTA data are summarized in Table 1. The pH of carbonated water increased from 4.23 to 4.28, 4.39, and 4.34 for the pressure cycling, sonication, and Tesla Valve methods, respectively.

None of the methods was able to create NBs directly in aqueous 50% MeOH solutions. However, once bubbles were created in 3% EtOH, they remained stable when diluted with methanol to produce the 50% MeOH spray solution and remained stable in solution for at least 7 days (Figure S3). The average size distribution of bubbles increased with the addition of ammonium salts; however, a significant number of the smallest bubbles remained. With the addition of formic acid into the CO_2_ NB-containing solution, bubbles below 350 nm were no longer observed, as shown in Figure S4. Solution pH and concentration of electrolytes have been shown to greatly affect NB size and surface charges.^29^ Formic acid addition can also lead to the formation of bicarbonate (HCO_3_^–^) ions from CO_2_.

### Effect of Nanobubbles on Small Molecules

**Figure 1:**
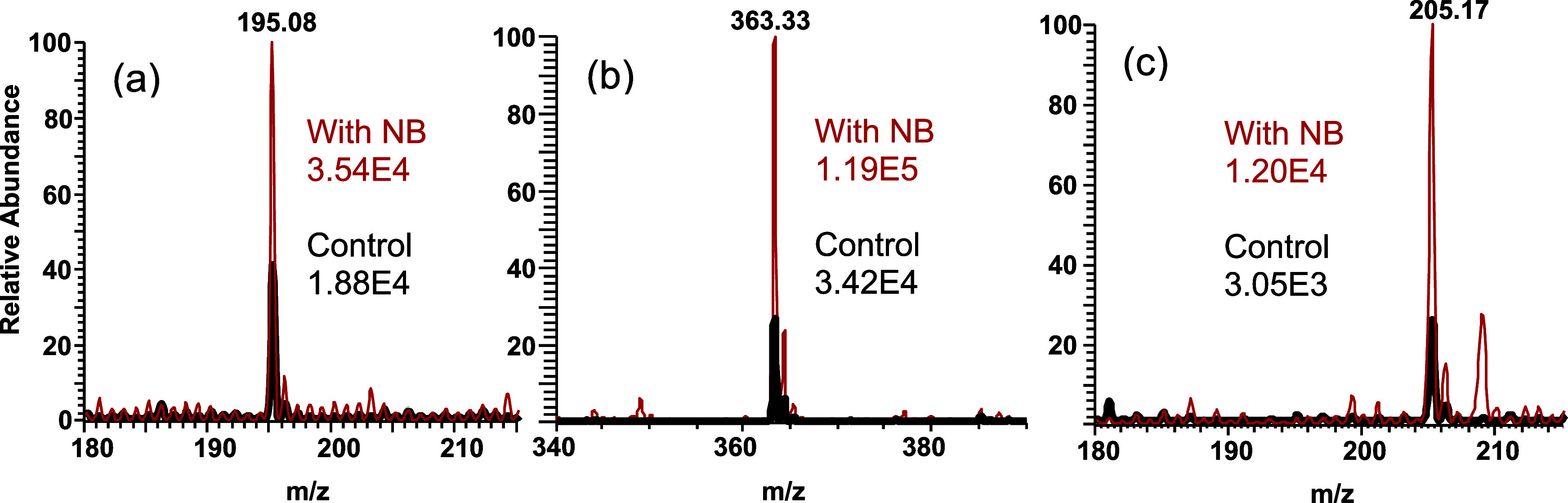
Representative spectra of (a) caffeine, (b) hydrocortisone, and (c) ibuprofen in 50% MeOH with CO_2_ NBs (red traces) and control without CO_2_ NBs (black traces). The intensities provided indicate the absolute signal intensity.

Hydrocortisone is a steroid hormone that is produced in the adrenal cortex. Caffeine is a stimulant and an effective analgesic adjuvant that can increase the antinociceptive effect of NSAIDs while reducing the probability of side effects.^30^ Both these molecules were clearly recognized as their [M + H]^+^ ions in the MS spectrum at *m*/*z* 363.3 and 195.1 for hydrocortisone and caffeine, respectively.^31^ The addition of CO_2_ and N_2_ NBs into the hydrocortisone- or caffeine-containing solutions resulted in significantly increased signal responses that were analyte and solution additive, as well as NB composition-dependent. Representative spectra of caffeine and hydrocortisone in 50% MeOH with and without CO_2_ NBs are shown in Figure 1a,b and with ammonium bicarbonate and formic acid in Figures S5C,E and S6C,E.

In 50% MeOH solutions, the caffeine signal nearly doubled (1.9×) with CO_2_ NB addition, while the hydrocortisone signal increased by 3.5 times. When 100 mM ammonium bicarbonate (ABC) was present in solution, the caffeine signal increased by a factor of 3 compared to that of pure solvent. However, when NBs were also present, the signal was further increased by another three times for a total increase of 9× relative to that of 50% MeOH (Figure S5C). The addition of ABC did not change the signal intensity of hydrocortisone relative to that of 50% MeOH, while the addition of CO_2_ NBs in the ABC-containing solution still increased the signal three times. The average signal intensities of caffeine and hydrocortisone in different solvent systems in the CO_2_ NB are shown in Figures S7 and S8.

Formic acid addition increased the signals of both compounds compared to the no-additive 50% MeOH controls by 2–4 times. CO_2_ NB addition, however, did not provide any signal enhancements when added to the FA-containing solution, as shown in Figures S5E and S6E.

Notably, using N_2_ NBs instead of CO_2_ with a 0.2% formic acid (FA) solvent system resulted in 2.4× improvement for caffeine and 1.8× improvement for hydrocortisone when compared to 0.2% FA in 50% MeOH:H_2_O as shown in Figures S5F and S6F. The average signal intensities of caffeine and hydrocortisone in the three different solvent systems with and without N_2_ NBs are shown in Figure S9.

In negative mode, ibuprofen, a popular analgesic and nonsteroidal anti-inflammatory drug,^32^ was clearly recognized as its [M–H]^−^ ion at *m*/*z* 205.2 in the mass spectrum. An improvement of 3.7× in signal intensity was observed when CO_2_ NB-containing solvent was added as the water fraction for 1 μM ibuprofen prepared in 50% MeOH. The representative spectra of ibuprofen in 50% MeOH with and without NBs are given in Figure 1c. The average signal intensities of ibuprofen in three different solvent systems with and without CO_2_ NBs are shown in Figure S10.

The signal intensities for a mixture of hop bittering acids in the ICE-4 hops extract were increased when CO_2_ NBs were added into the water fraction of this analysis. What is remarkable here is that a 1.6× improvement was observed across the four major compounds, even as the water fraction containing NBs was only 5% of the total, as shown in Figure S11.

Bubbles produced by all three methods improved the signal intensities, although there were differences in the magnitude of the enhancement effect obtained by the three generation methods. The average signal intensities of caffeine and ibuprofen with and without CO_2_ NBs using the three different NB generation methods are shown in Figures S12 and S13. In positive mode with CO_2_ NBs, similar improvements were observed when no additive was present with all three generation methods. When ammonium bicarbonate was also present in the final working solution, NBs produced by Tesla valve flow switching increased the signal significantly more. When formic acid was used as an additive, signal increases were only observed with CO_2_ NBs produced by pressure cycling. In negative mode, increases were large for the 50% MeOH solvent systems, especially with the pressure cycling and Tesla valve flow switching methods, and less when sonication was used. As discussed above, when NBs were generated from N_2_ by the Tesla valve, the signal was also improved with both ABC- and FA-containing solvent systems, as shown in Figures S5D,F and S6D,F.

These differences in enhancement factors, observed for NBs produced with the different methods, cannot be attributed to the physical characterization of the produced NBs shown in Table 1 and will be investigated in future work.

It is clear that NBs increase the signal response in ESI-MS in both positive and negative modes for small molecules in a variety of solvent systems. Furthermore, NBs when used in ESI do not cause any notable changes in signal stability. For the results shown in Figure S14, the relative standard deviation for ESI was 8.3% without NB and 7.5% with NBs.

### Ion Suppression

**Figure 2:**
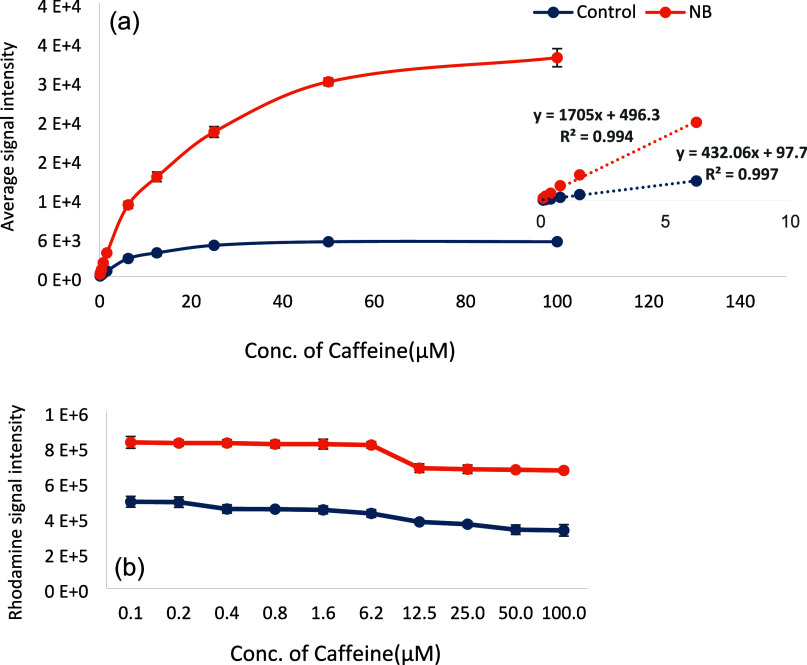
Average signal intensities of (a) caffeine with (orange traces) and without (blue traces) CO_2_ NBs and (b) Rhodamine signal with increasing caffeine concentration with CO_2_ NBs (orange trace) and without (blue trace) in a 50% MeOH solvent system.

Quantitative analysis using ESI-MS relies on the correlation between the mass spectral response and the concentration of the analyte.^33,34^ However, this method faces challenges in the case of complex mixtures due to ion suppression, where the presence of other charged species reduces analyte signals.^35−37^ As the concentrations of interfering substances increase, the analyte signals are more significantly suppressed, making it difficult to accurately quantify a specific compound in a mixture.^38^ The mass and charge of analytes also influence their susceptibility to ion suppression or their potential to cause it for other compounds. Ion suppression is a consequence of competition for excess charge near the droplet surface, where analytes with more hydrophobicity tend to reside.^39^ More polar analytes are more susceptible to suppression, while organic solvents generally enhance the ESI signal, especially in positive-ion mode.^40^ To study ion suppression effects in the analysis of small analyte mixtures by ESI-MS, a double-component mixture with and without CO_2_ NBs was used. The two model analytes investigated were Rhodamine 6G and caffeine. Ion suppression of Rhodamine 6G by caffeine is shown in Figure 2b for solutions with and without NBs. The intensity of the constant concentration of the Rhodamine 6G signal decreases as the concentration of caffeine increases in the control solution. However, when NBs are present, the Rhodamine 6G signal remains constant beyond 6 μM caffeine concentration, showing a significantly reduced ion suppression effect.

The calibration curves of caffeine with and without NBs are shown in Figure 2a. The limit of detection and limit of quantification are calculated from the slope and the standard deviation of the signal, respectively.^41,42^ The LODs and LOQs are given in Table 2.

**Table 2:** LOD and LOQ Values in the 50% MeOH Solvent System

solvent system	**LOD (μM)**	**LOQ (μM)**
50% MeOH:H_2_O	0.2	0.74
50% MeOH:NBs	0.04	0.15

### Effect of NBs on Proteins

**Figure 3:**
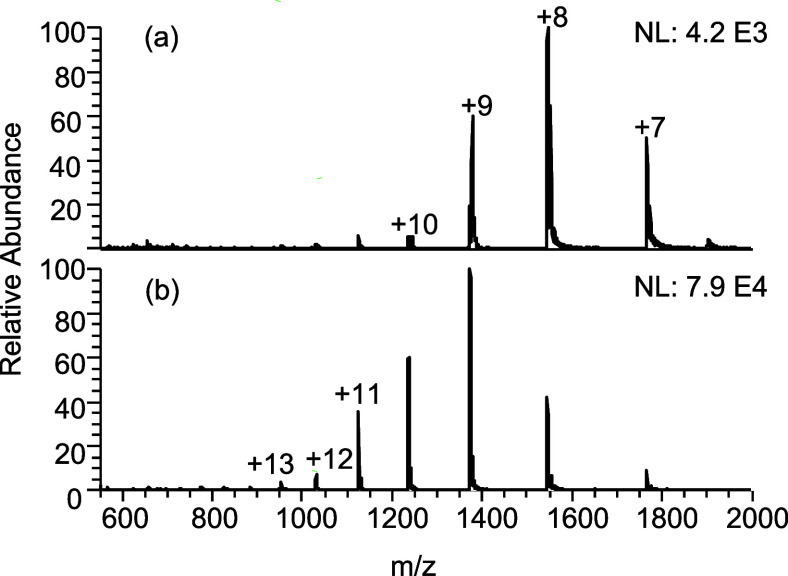
Representative ESI-MS spectra of 10 μM cytochrome c in (a) 100% water and (b) 100% water containing CO_2_ NBs. The normalization level (NL) indicates the absolute signal intensity.

Cytochrome c is a small heme protein found in the mitochondria of eukaryotic cells. It plays a crucial role in the electron transport chain, which is part of cellular respiration.^43^ The protein cytochrome c is in its native state in 100% H_2_O as shown in Figure 3a. Based on multiple studies, including ion mobility mass spectrometry measurements, generally, cytochrome c charge states ranging from +9 to +6 represent folded protein conformations, whereas charge states above +9 represent different degrees of extended, unfolded protein conformations.^44^ We observed that the highest intensity charge state (HICS) of cytochrome c in 100% H_2_O is +8, as shown in Figure 3a.

Analyzing cytochrome c in a CO_2_ NB-enriched solvent system, a dramatic increase in protein signal is observed (Figure 3b). Here, the intensity of the HICS increased by a factor of 18.7. In addition to the dramatic increase in signal intensity, the charge envelope shifts to higher charge states, indicative of protein unfolding. The average charge state increases from *z* = +8.1 to *z* = +9.6. The incorporation of CO_2_ NBs to a protein solution indeed causes unfolding of protein during the electrospray as previously hypothesized,^7^ although not to the same extent as in electrothermal supercharging.^45^ Complete unfolding required the presence of ammonium salts as shown in a subsequent study.^52^

### Effect of Nanobubbles on Accelerating Microdroplet Reactions

Lately, there has been a keen interest in the acceleration of chemical reactions in microdroplets.^46−49^ Several factors that cause accelerated reactions in microdroplets are proposed, such as partial solvation of reagent ions in microdroplets, ordered orientation of solutes and solvents, fast solvent evaporation, which causes an increase in the concentration of reagent solution, increased surface-to-volume ratio, increasing mass transfer efficiency, and fast mixing and diffusion.^49^

NBs have the ability to further increase the reaction rates by also increasing the liquid air interface surface area, albeit here internal to the droplet. Further, NBs have many features that could also lead to increased reaction rates, including high field strengths, high mass transfer efficiency, and increased solubility of gases in liquids due to the high internal pressures.^50,51^

**Figure 4:**
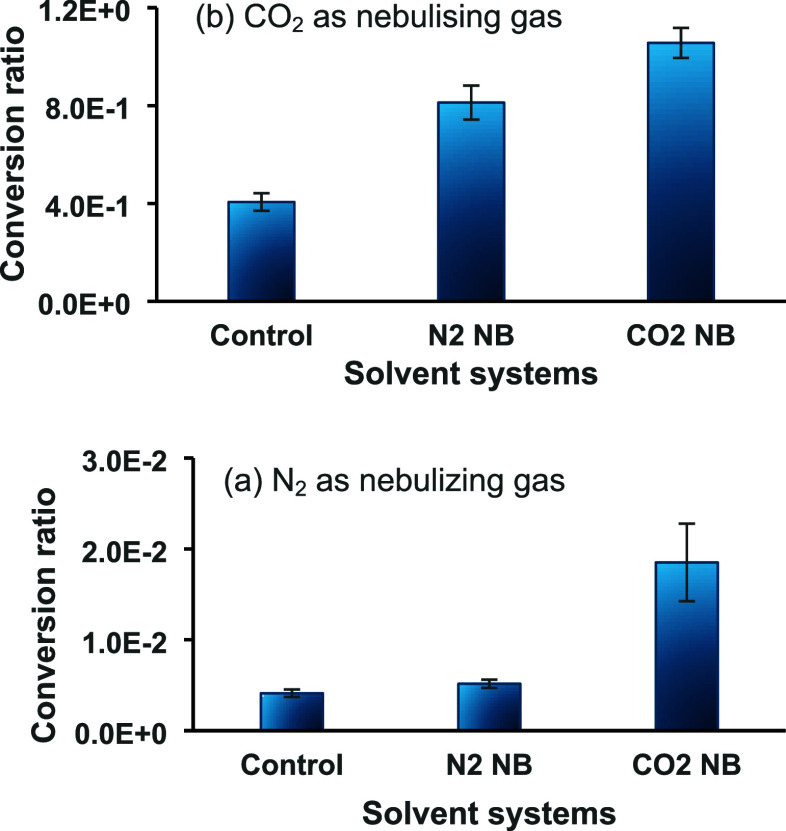
Conversion ratio of 10 μM *N*,*N*-dibutyl-1,3-propane diamine (DBPA) in different solvent systems when using (a) N_2_ as the nebulizing gas and (b) CO_2_ as the nebulizing gas. Conversion ratio is calculated as the ratio of intensity of the product ion over that of the reagent ion.

To demonstrate the ability of NBs to further accelerate microdroplet reactions, we use the reaction between a diamine, *N*,*N*-dibutyl-1,3-propane diamine (DBPA), and carbon dioxide as a model reaction. The amine–carbon dioxide chemistry is significant due to its importance in the CO_2_ capture industry. Recently, it has been shown that when CO_2_, which is a reagent itself, is used as the nebulizing gas, the conversion rate of DBPA to protonated DBPA carbamic acid can be significantly increased over using traditional N_2_ nebulization while relying only on dissolved atmospheric CO_2_.^48^ The published increase in conversion ratio is confirmed with the results presented below in Figure 4, where the conversion ratio increased by 98 times when switching from N_2_ to CO_2_ nebulizing gas. However, when N_2_ NBs are also added into the solution, the conversion rate is further doubled. Since CO_2_ is one of the reactants in this scheme, when the NBs are composed of CO_2_, an additional increase in conversion ratio is observed. Here, the NB effect is further augmented by overcoming the solubility barrier by delivering the reactant at high concentration to the bulk solution and creating significantly larger liquid–gas interface area.

Carbon dioxide NBs also provide an increase in conversion ratio when N_2_ is used as a nebulizing gas. Here, the addition of CO_2_ NBs to the spray solvent leads to an increase in the conversion ratio of 4.5× compared to relying on atmospheric CO_2_ alone. It should be noted that even when relying only on atmospheric CO_2_, in the presence of N_2_ NBs in the sample solution, a 1.25× improvement in conversion ratio was achieved compared to when no NBs were present.

The results in Figure 4 show that there are multiple benefits to adding NBs into reaction mixtures that depend on activation by their chemicophysical properties, and as effective reactive gas delivery vehicles, overcoming the solubility barrier.

## Conclusions

The simple addition of NBs into spray solvents is shown to significantly improve signal responses of small molecules and a protein in both positive and negative modes during ESI-MS. This leads to lower limits of detection. Furthermore, ion suppression is reduced somewhat, extending the limits of linearity. The combined effect produces a wider linear dynamic range. The presence of NBs in spray solvents also unfolds protein molecules, as demonstrated for cytochrome c. This is tentatively attributed to the NBs increasing the total area of the hydrophobic gas–liquid interface onto which proteins can unfold, as previously suggested.^7^ This increased interface area improves reaction kinetics and enhances gas transfer and is also a contributing factor in the further enhancement in the conversion ratios when NBs are present in solution compared to those measured from standard reactive-ESI experiments. Moreover, NBs can overcome solubility barriers when one of the reagents is gaseous and thus can be incorporated into an NB. This is demonstrated in the reaction between *N*,*N*-dibutyl-1,3-propane diamine and carbon dioxide, where conversion rates of the reaction can be significantly improved when N_2_ NBs are present in solution and even more so when the NBs are composed of CO_2_. Interestingly, NBs generated using different methods of preparation affect the mass spectra differently, implying that mass spectrometry may offer a novel perspective on NB chemistry that is not captured through current characterization methods.

## Supporting Information Available

Experimental setup of the Tesla valve NB generator. Size distribution and stability of CO_2_ bubbles in different methods and solvent systems. Mass spectra and average signal intensity graphs of small molecules in both CO_2_ and N_2_ bubbles in different solvent systems. Signal stability with and without nanobubbles (PDF)
