# Supplementary material for: Nanobubbles in Electrospray Ionization Mass Spectrometry
Source: Anal Chem. 2025 Mar 10;97(11):6034–40. doi: 10.1021/acs.analchem.4c06040 (PMC11948179; doi:10.1021/acs.analchem.4c06040)
Supplement: Supplementary file 1 — ac4c06040_si_001.pdf [file ac4c06040_si_001.pdf]

Nanobubbles in Electrospray Ionization Mass Spectrometry

George Joseph, Bincy Binny, Andre R Venter*

Department of Chemistry, Western Michigan University, Kalamazoo, Michigan, US, 49008-5413

andre.venter@wmich.edu

Table of Contents

Figure S1. Experimental Setup of Tesla Valve NB Generator

Figure S2. Size distribution determined by dynamic light scattering (DLS) of CO_2 nanobubbles created from carbonated 3% Ethanol in water using (A) flow regime switching by Tesla valve, (B) Pressure cycling, and (C) Sonication.

Figure S3. Stability of CO_2 NBs diluted in 50% MeOH (A) Day1, (B) Day7. Diameters were determined by DLS.

Figure S4. Size distribution of CO_2 bubbles in (A) 100mM ABC and (B) 0.2% FA

Figure S5. Representative spectra of caffeine in 50% MeOH (A), 100mM ABC (C), 0.2% FA (E) with (red traces) and without (black traces) CO_2 NBs and 50% MeOH (B), 100mM ABC (D), 0.2%FA (F) with (red traces) and without (black traces) N_2 NBs.

Figure S6. Representative spectra of hydrocortisone in 50% MeOH (A), 100mM ABC (C), 0.2% FA (E) with (red traces) and without (black traces) CO_2 NBs and 50% MeOH (B), 100mM ABC (D), 0.2%FA (F) with (red traces) and without (black traces) N_2 NBs.

Figure S7. Average signal intensity of caffeine in three different solvent system with and without CO_2 NBs.

Figure S8. Average signal intensity of hydrocortisone in three different solvent system with and without CO_2 NBs.

Figure S9. Average signal intensity of ibuprofen in three different solvent system with and without CO_2 nanobubbles.

Figure S10. Average signal intensity of (A) caffeine, (B) hydrocortisone in three different solvent system with and without N_2 NBs.

Figure S11. Representative spectra of Hops acid in 95%MeOH with NBs (red traces) and the control without NBs (black traces).

Figure S12. Average signal intensity of caffeine with and without NBs using three different NB generation methods.

Figure S13. Average signal intensity of ibuprofen with and without NBs using three different NB generation methods.

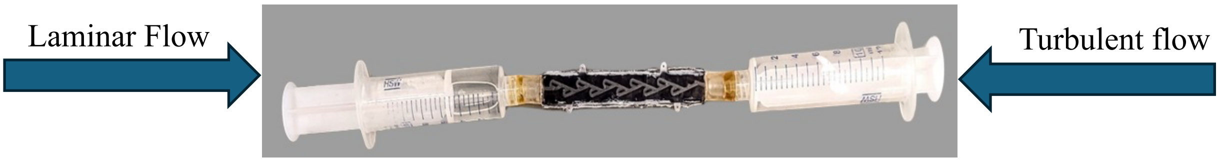

Fig. S1. Experimental Setup of Tesla Valve Nanobubble Generator. A Tesla Valve, the 3-D printed black device between the two syringes, is a valvular conduit without moving parts. Flow from the left in unimpeded and laminar, while flow from the right experiences turbulence at low Reynolds Numbers.

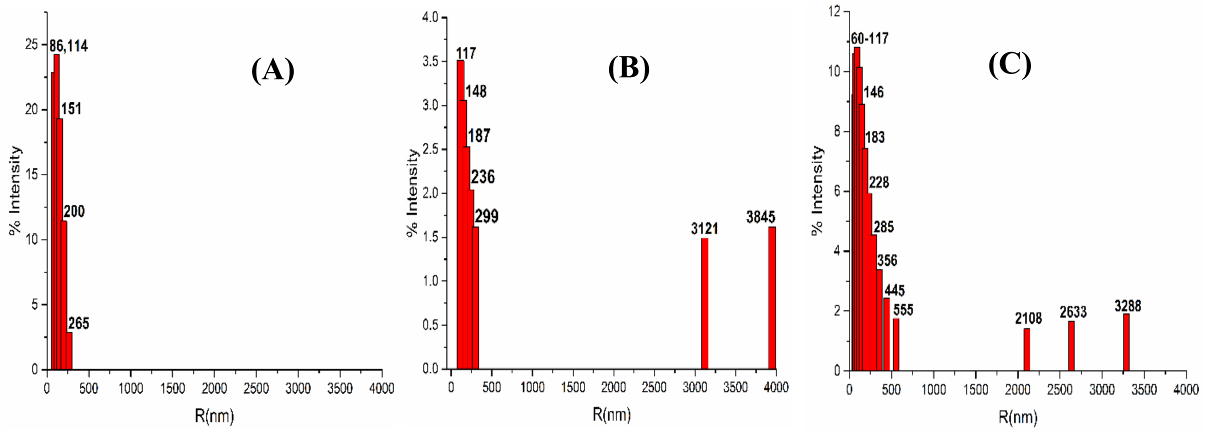

Fig. S2. Size distribution determined by dynamic light scattering (DLS) of CO_2 nanobubbles created from carbonated 3% Ethanol in water using (A) flow regime switching by Tesla valve, (B) Pressure cycling, and (C) Sonication.

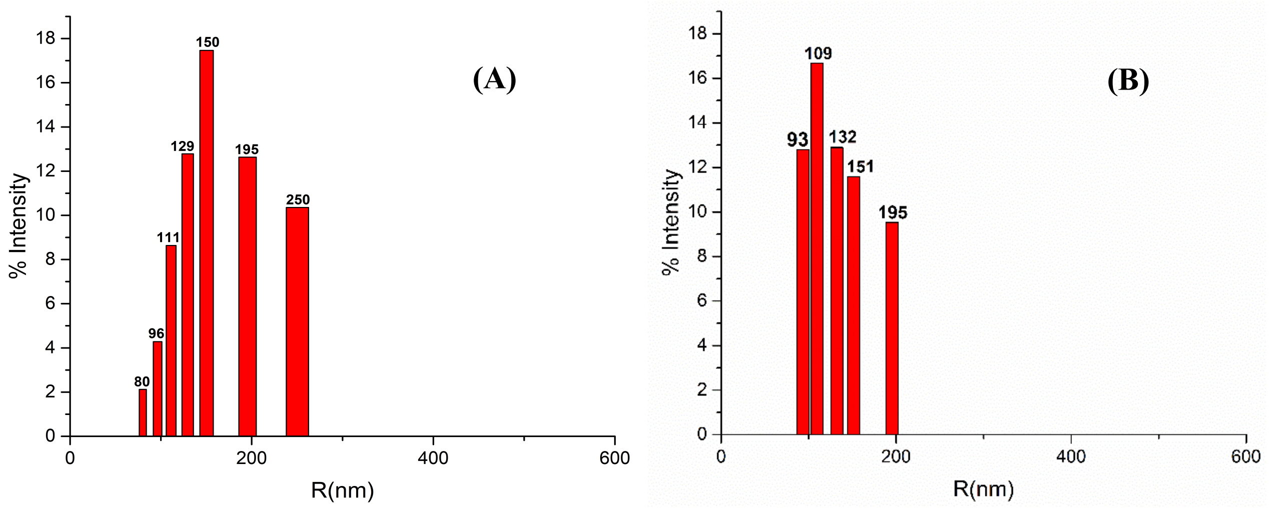

Fig. S3. Stability of CO_2 NBs diluted in 50% MeOH (A) Day1, (B) Day7. Diameters were determined by DLS.

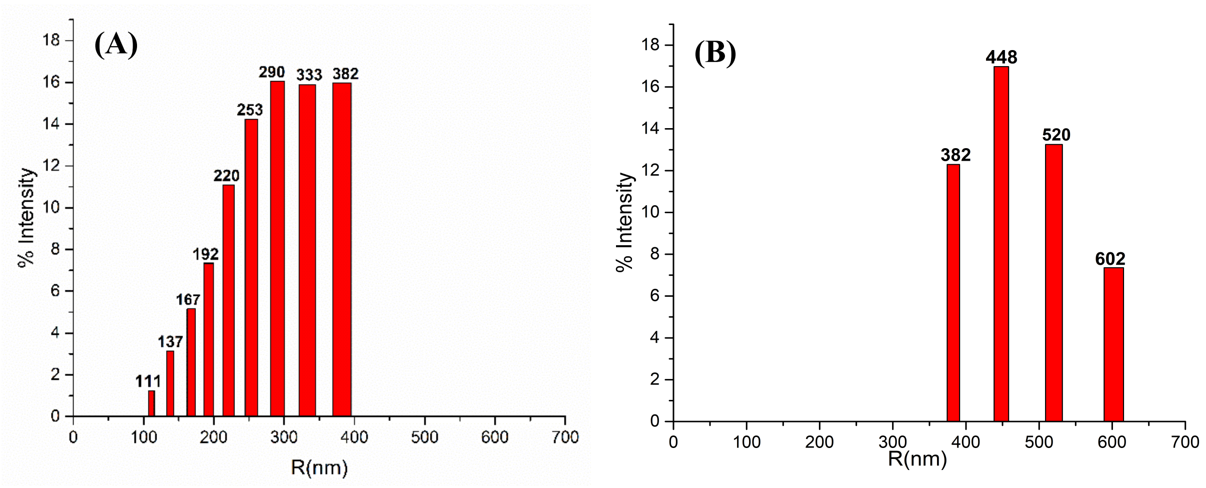

Fig. S4. Size distribution by DLS of CO_2 bubbles in (A) 100mM ABC and (B) 0.2% FA.

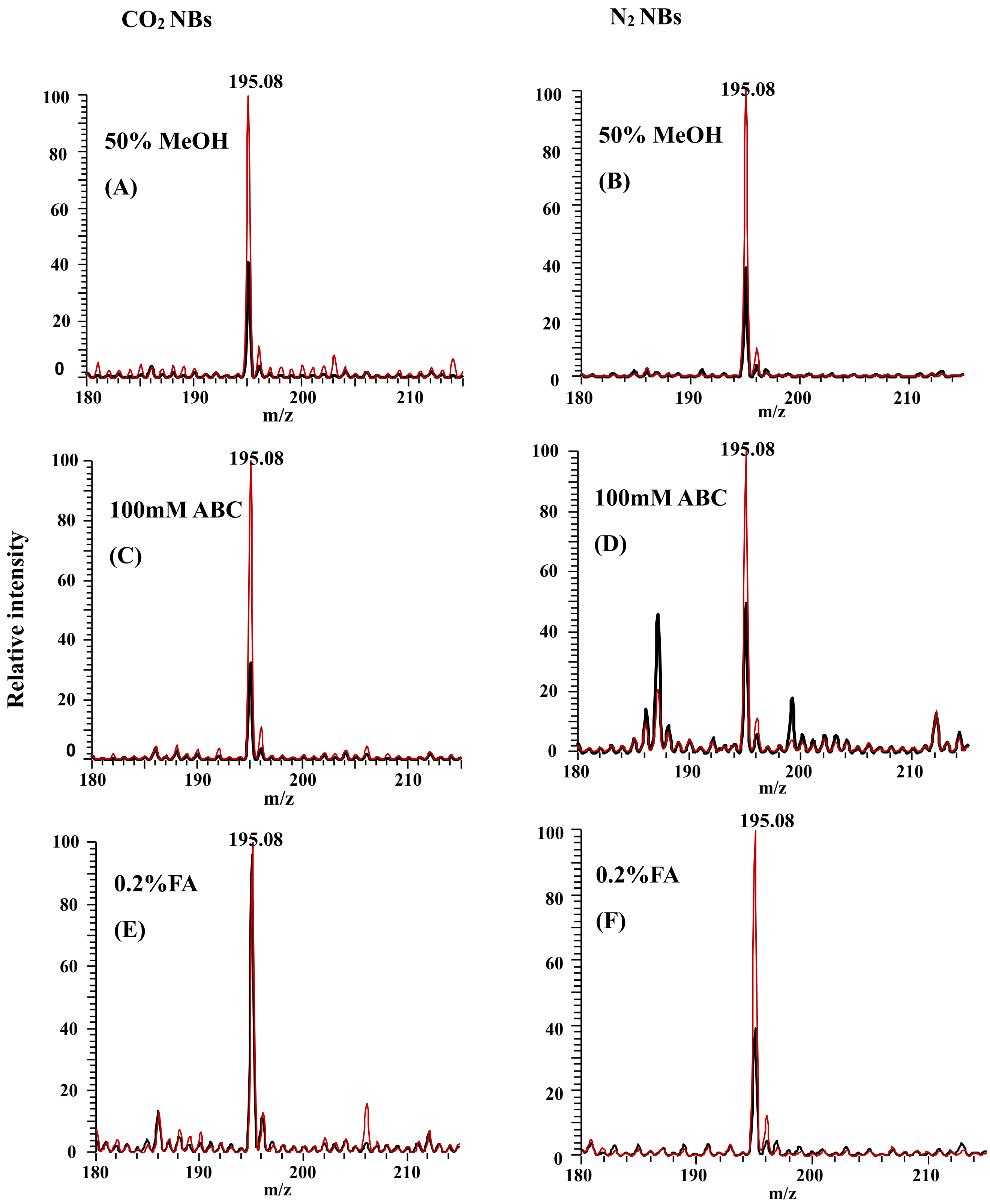

Fig. S5. Representative spectra of caffeine in 50% MeOH (A), 100mM ABC (C), 0.2% FA (E) with (red traces) and without (black traces) CO_2 NBs and 50% MeOH (B), 100mM ABC (D), 0.2%FA (F) with (red traces) and without (black traces) N_2 NBs.

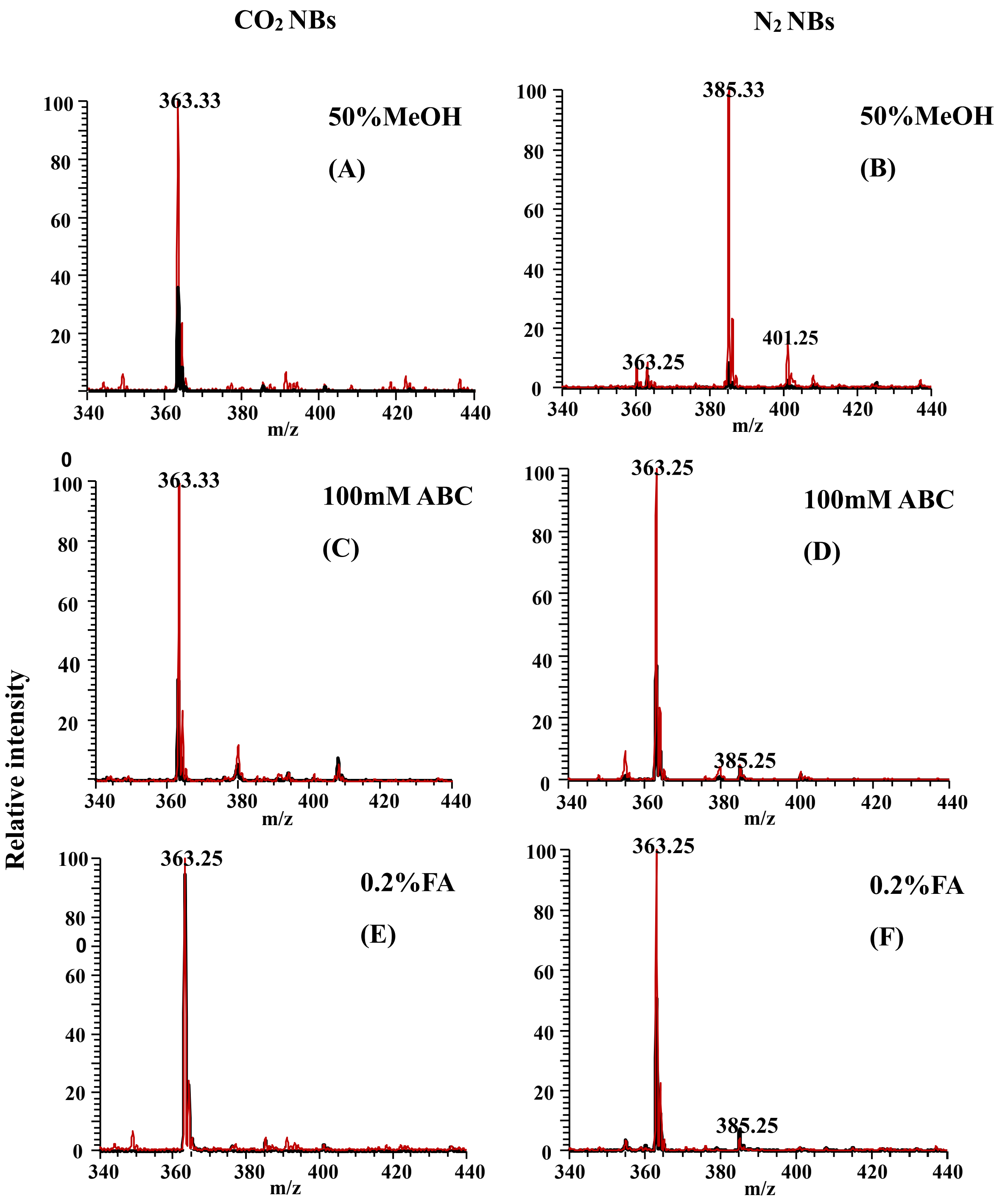

Fig. S6. Representative spectra of hydrocortisone in 50% MeOH (A), 100mM ABC (C), 0.2% FA (E) with (red traces) and without (black traces) CO_2 NBs and 50% MeOH (B), 100mM ABC (D), 0.2%FA (F) with (red traces) and without (black traces) N_2 NBs.

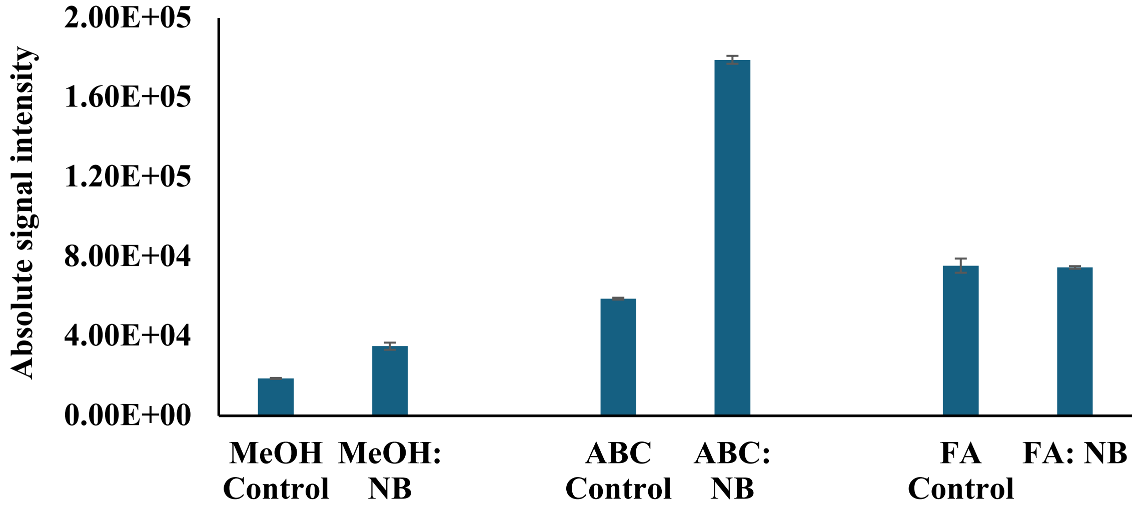

Fig. S7. Average signal intensity of caffeine in three different solvent system with and without CO_2 NBs.

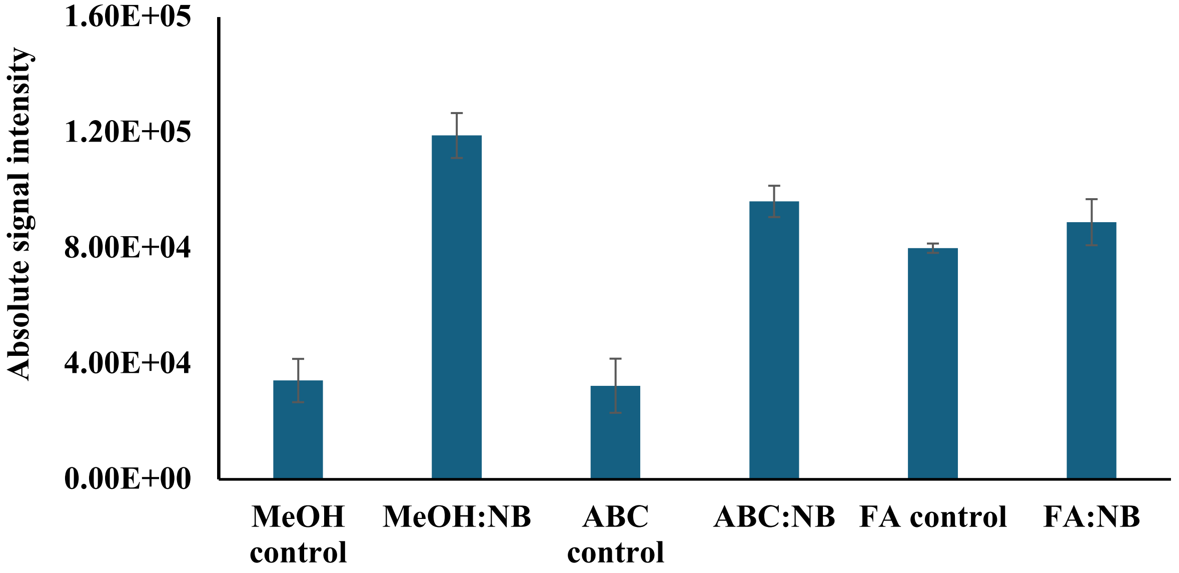

Fig. S8. Average signal intensity of hydrocortisone in three different solvent system with and without CO_2 NBs.

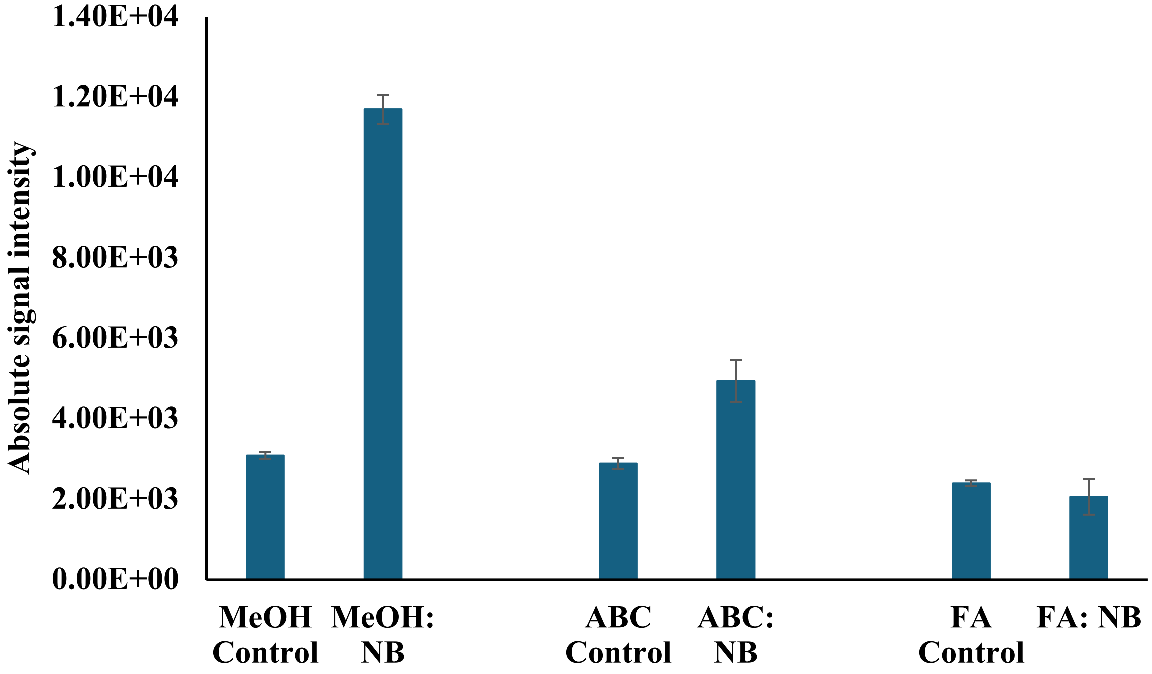

Fig. S9. Average signal intensity of ibuprofen in three different solvent system with and without CO_2 NBs.

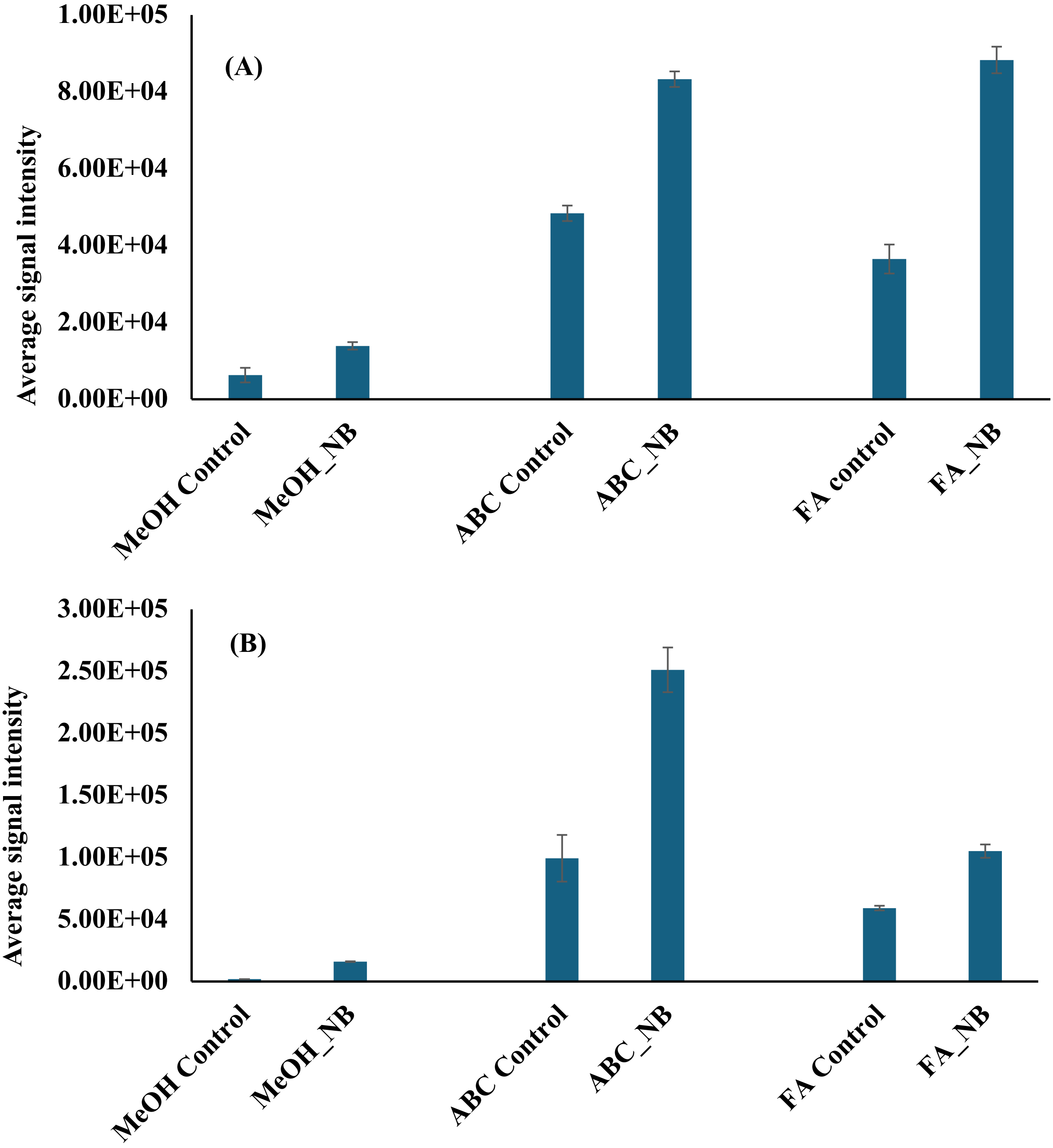

Fig. S10. Average signal intensity of (A) caffeine, (B) hydrocortisone in in three different solvent system with and without N_2 NBs.

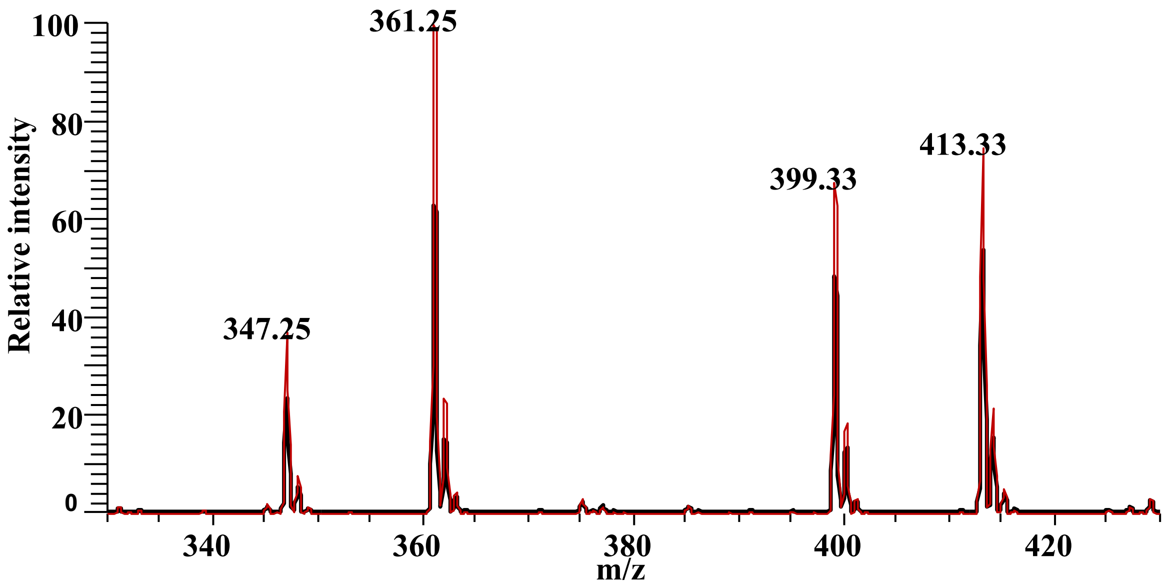

Fig. S11. Representative spectra of Hops acid in 95%MeOH with NBs (red traces) and the control without NBs (black traces).

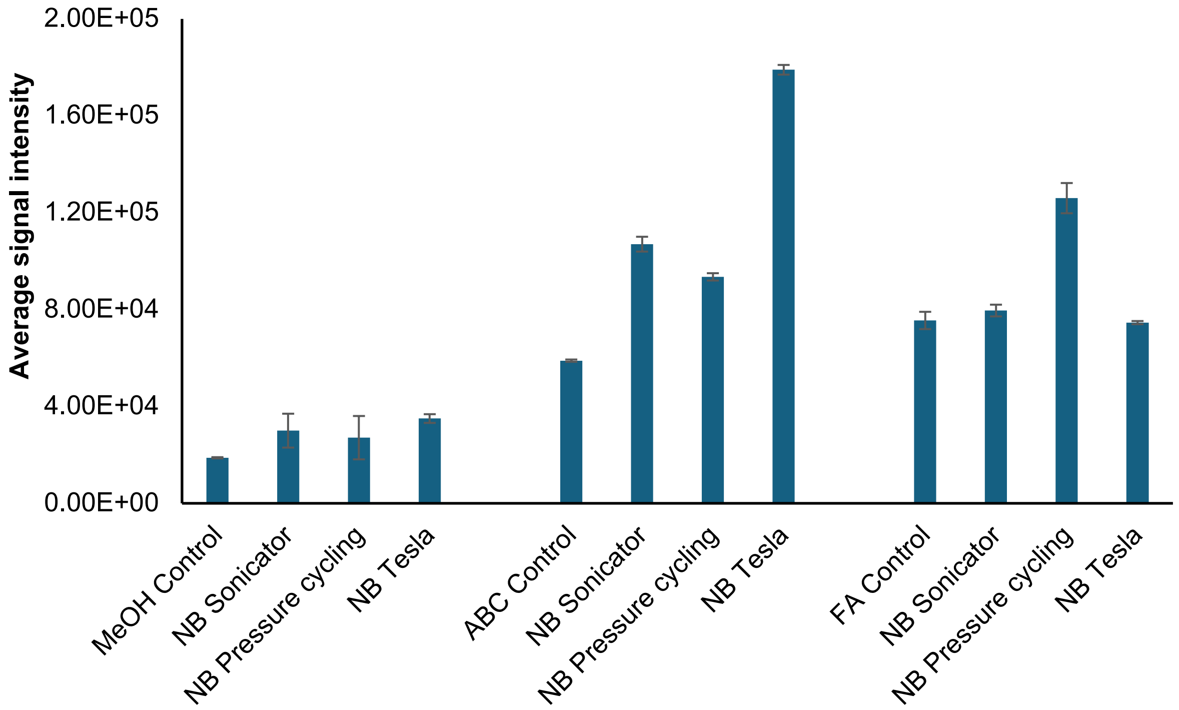

Fig. S12. Average signal intensity of caffeine with and without CO_2 NBs using three different NB generation methods.

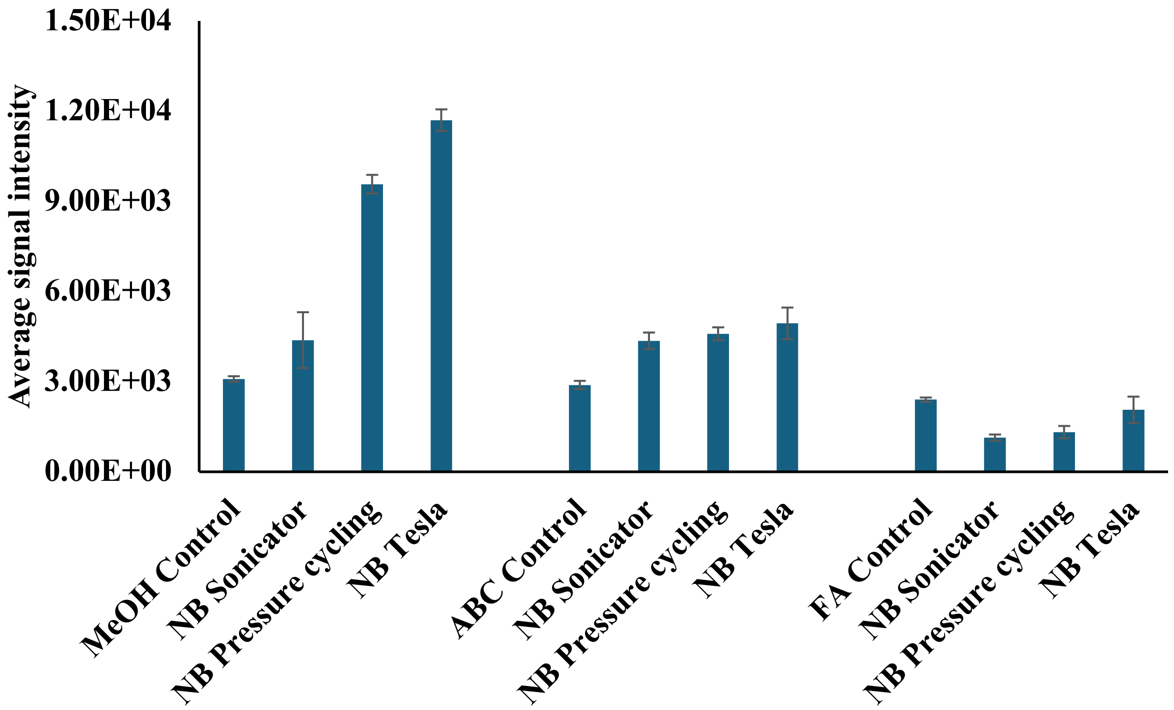

Fig. S13. Average signal intensity of ibuprofen with and without NBs using three different NB generation methods.

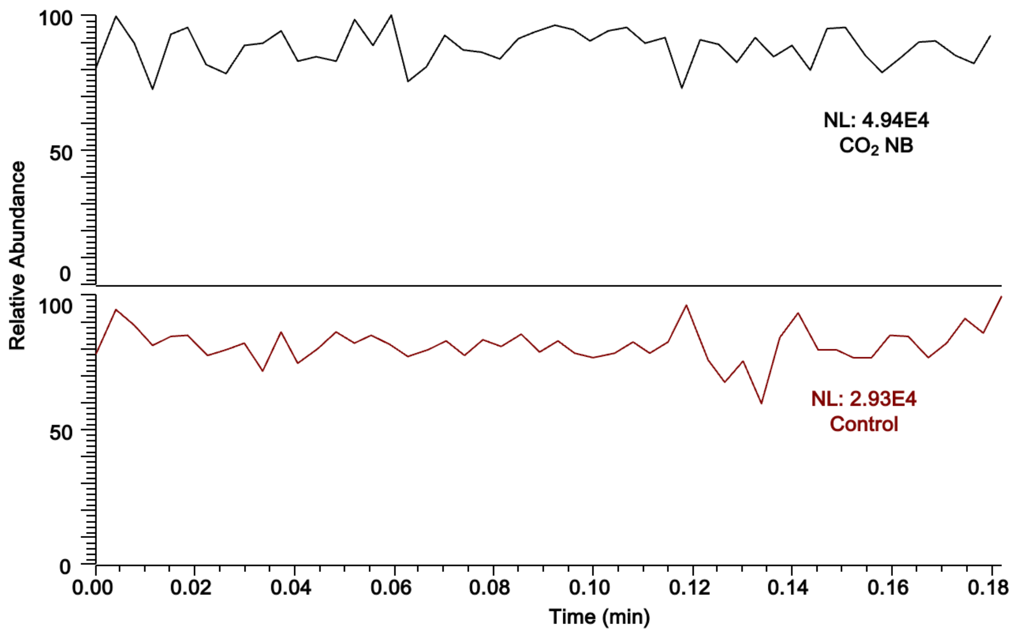

Fig. S14. Signal Stability of ESI with and without NBs.
